# Curcumin supplementation attenuates the decrease in endothelial function following eccentric exercise

**DOI:** 10.20463/jenb.2019.0010

**Published:** 2019-06-30

**Authors:** Youngju Choi, Yoko Tanabe, Nobuhiko Akazawa, Asako Zempo-Miyaki, Seiji Maeda

**Affiliations:** 1 Faculty of Health and Sport Science, University of Tsukuba, Tsukuba Japan; 2 Department of Sport Research, Japan Institute of Sports Sciences, Tokyo Japan; 3 Faculty of Sports and Health Sciences, Ryutsu Keizai University, Ryugasaki Japan

**Keywords:** supplementation, endothelial function, curcumin, flow-mediated dilation, eccentric exercise

## Abstract

**[Purpose]:**

Eccentric exercise induces a decrease in vascular endothelial function. Curcumin, a major component of turmeric, has potent antioxidant and anti-inflammatory properties that are associated with vascular protective effects. The present study examined the effect of acute supplementation of curcumin on eccentric exercise-induced endothelial dysfunction in healthy young men.

**[Methods]:**

Fourteen healthy sedentary young men (range, 21–29 years) were assigned to either the curcumin (n = 6) or placebo (n = 8) group. All subjects consumed either curcumin or placebo before exercise, and eccentric exercise of the elbow flexors was performed with their nondominant arm. Before and 60 min after exercise, brachial artery flow-mediated dilation (FMD), as an indicator of endothelial function, was measured in the non-exercised arm.

**[Results]:**

Brachial artery FMD significantly decreased following eccentric exercise (*p* < 0.05) in the placebo group, but acute supplementation with curcumin before exercise nullified this change. The change in FMD before and after eccentric exercise between the placebo and curcumin groups was significantly different (*p* < 0.05).

**[Conclusion]:**

The present study found that acute curcumin supplementation could attenuate the decrease in endothelial function, as measured by FMD, following eccentric exercise in healthy young men.

## INTRODUCTION

Resistance exercise is known to have beneficial effects in the musculoskeletal system and widely recommended for preventive and rehabilitative programs of physical activity by major professional health organizations^1^. Resistance exercise incorporates a combination of concentric, eccentric, and isometric muscle contractions. Eccentric exercise is a more effective method in improving muscle strength^2^ and often used by athletes and other individuals. 

Eccentric exercise is associated with increased oxidative stress and inflammation^3-5^, both of which impair endothelial function^6^. Endothelial function is commonly evaluated by brachial artery flow-mediated dilation (FMD)^7,8^. Recently, we observed decreased FMD in sedentary subjects performing eccentric exercise^9^, as well as in those performing other types of resistance exercise^10,11^. Decreased FMD is an early hallmark of cardiovascular disease and a strong predictor of future cardiovascular events^12^. Therefore, despite the favorable effects of eccentric exercise in the musculoskeletal system, interventions are necessary to attenuate a decrease in endothelial function following eccentric exercise.

Curcumin, a commonly used spice and a yellow pigment in food, is a major active component of turmeric. Curcumin has potent antioxidant and anti-inflammatory properties^13^. A previous study from our laboratory and other studies have shown that chronic curcumin consumption in humans protects against vascular endothelial dysfunction^14,15^. However, these studies were limited to chronic effects of curcumin on endothelial function in postmenopausal older women or young healthy subjects. No known studies have assessed whether acute curcumin supplementation can improve the decrease in endothelial function induced by eccentric exercise. Therefore, the present study aimed to examine whether acute curcumin supplementation would attenuate a decrease in eccentric exercise-induced endothelial function in healthy young men. We hypothesized that acute curcumin supplementation would result in a protective effect against the decrease in endothelial function following eccentric exercise.

## METHODS

### Subjects

Fourteen healthy young men (age, 21–29 years) volunteered to participate in this study. All subjects had not been involved in any regular resistance training at least 1 year before this study. They were normotensive and nonobese, and none exhibited signs or symptoms or had a history of any chronic diseases. Furthermore, all subjects were non-smokers, and none of them were using any medications, anabolic steroids, or antioxidant-containing dietary supplements during the study. The subjects were assigned to a group that performed eccentric exercise, in which they consumed either a placebo (n = 8) or curcumin (n = 6) before performing eccentric exercise. The present study was approved by the Ethical Committee of the University of Tsukuba. This study conformed to the principles outlined in the Declaration of Helsinki, and all subjects provided written informed consent before participation in this study.

### Experimental protocol

Before the experiment, the subjects refrained from alcohol and caffeine intake for at least 12 h and intense physical activity (exercise) for at least 72 h to rule out any possible acute effect. On the day of the experiment, the subjects rested in the supine position for a minimum of 15 min in a quiet, temperature-controlled room (25°C–26°C). With the subject in the supine position, resting brachial blood pressure, heart rate, and brachial endothelial function were measured. In the sitting position, blood sampling was performed to determine metabolic risk factors. After these measurements, the subjects ingested either 150 mg curcumin in a pill form or 150 mg placebo in an identical pill form. Subsequently, each subject performed eccentric exercise using their nondominant elbow. The pre-exercise measurements (brachial blood pressure, heart rate, and brachial endothelial function) were repeated 60 min after exercise.

### Supplementation

The curcumin group orally ingested six capsules of 25 mg curcumin (150 mg), which consists of highly absorptive curcumin dispersed with colloidal nanoparticles (Theracurmin; Theravalues Corporation, Tokyo, Japan). Similarly, the placebo group ingested six placebo capsules, which were similar in shape and color and contained equivalent does of starch (dextrin and maltose). A previous study has shown that the plasma concentrations of curcumin increased 1–2 h after ingestion^16^. Therefore, the participants in this study were provided curcumin or placebo capsules 1 h before exercise. 

### Eccentric exercise

The subjects performed 1 set of 50 repetitions of an elbow flexor eccentric exercise by using a Biodex isokinetic dynamometer (Biodex System 3, Sakai Medical Co., Ltd., Japan), as previously described^9,17^. The nondominant arm was selected for exercise to minimize the effects on daily life. The subjects were seated on the dynamometer chair with the arm supported and were stabilized at the waist and chest. Starting with the elbow flexed to 50° and ending at an angle of 170°, each subject performed 1 set of 50 maximal eccentric movements of the elbow flexor, at an angular velocity of 120°·s^-1^. The subjects were verbally encouraged to produce maximal effort to resist elbow extension by the dynamometer. The subjects were provided a 12-s rest between each contraction, during which time the dynamometer arm returned passively to the starting position. Total work and peak torque were calculated using the Biodex System software.

### Resting blood pressure and heart rate

Systolic and diastolic blood pressures (mmHg) and heart rate were measured from the non-exercised arm in the supine position by oscillometry and echocardiogram (Form PWV/ABI; Colin Medical Technology, Japan). 

### FMD

Endothelial function was evaluated noninvasively using brachial artery FMD. Brachial artery FMD was measured using a stereotactic probe-holding device equipped with an edge-tracking system for 2D imaging and pulsed Doppler flow velocimeter for automatic measurement (UNEXEF; Unex Co. Ltd., Japan), as previously described^9^. Briefly, the diameter of the non-exercised brachial artery at baseline was measured in place 5–10 cm proximal to the antecubital fossa using a 10-MHz linear array transducer probe. Subsequently, the cuff distal to the measurement site was inflated to 50 mmHg above the systolic blood pressure for 5 min and deflated. The diameter at the same point of the artery was monitored continuously for 3 min following deflation of the cuff, and the maximum dilation after deflation was recorded. FMD was calculated as the percent changes in the arterial diameter over the baseline value at maximal dilation after cuff deflation. FMD (%) = (maximal diameter - baseline mean diameter) / baseline mean diameter × 100.

### Blood sampling and analyses 

Blood was obtained from the antecubital vein using a standard venipuncture technique. The blood samples were centrifuged at 3000 rpm for 15 min at 4°C, and the serum and plasma samples were stored at -80°C until analysis. Serum cholesterol and plasma glucose concentrations were determined using standard enzymatic techniques. Plasma curcumin concentration was measured using HPLC–MS/MS system, consisting of a Prominence micro-C system (Shimadzu, Kyoto, Japan) and API 3200 tandem mass spectrometer (Applied Biosystems CA, USA). 

### Statistical analysis

The FMD between groups were compared using repeated-measures analysis of variance (ANOVA). Whenever a significant interaction for FMD was detected, t-tests were used to analyze individual differences between time points (paired). Furthermore, the comparison for the FMD between groups was assessed using analysis of covariance (ANCOVA) to correct the influence of baseline brachial artery diameter on FMD. Change in FMD response from pre- to post-exercise between groups was compared using unpaired t-test. Hemodynamic characteristics (blood pressure, heart rate, and, brachial artery diameter) were compared using repeated-measures ANOVA. Changes in the plasma curcumin concentration over time were compared between the curcumin and placebo groups using two-way repeated ANOVA. When the ANOVA showed a significant interaction effect, Bonferroni’s post hoc test was used for significant differences. The subject’s physiological characteristics (age, body mass index [BMI], cholesterol concentration, and glucose concentration) and total work and mean eccentric peak torque during the exercise were compared using unpaired t-test. All statistical analyses were performed using IBM SPSS Statistics 22 (SPSS Inc., Chicago, IL, USA). Values were expressed as mean ± SE, and a P-value < 0.05 was considered statistically significant.

## RESULTS

The subject characteristics are presented in Table 1. There were no significant differences in age; BMI; serum concentrations of total cholesterol, high-density lipoprotein (HDL) cholesterol, and low-density lipoprotein (LDL) cholesterol; and plasma concentration of glucose between the curcumin and placebo groups. 

**Table 1.** Characteristics of subjects

	Placebo (n = 8)	Curcumin (n = 6)
Age, years	23 ± 1	24 ± 1
Body mass index, kg/m^2^	22 ± 1	22 ± 1
Total cholesterol, mg/dL	168 ± 8	159 ± 8
HDL cholesterol, mg/dL	56 ± 3	52 ± 4
LDL cholesterol, mg/dL	100 ± 7	97 ± 9
Glucose, mg/dL	84 ± 2	87 ± 2

Values are presented as mean ± SE.

The total work (2875 ± 260 J vs. 2555 ± 349 J, *P* = 0.551) and mean peak torque (39 ± 3 Nm vs. 36 ± 4 Nm, *P *= 0.607) during eccentric exercise did not differ between the placebo and curcumin groups. 

The hemodynamic parameters before and after exercise are presented in Table 2. There were no differences in the pre-exercise systolic blood pressure, diastolic blood pressure, heart rate, and brachial artery diameter between the two groups. Furthermore, no changes were observed in these values from baseline for either group. 

**Table 2.** Hemodynamic parameters before and after eccentric exercise

	Placebo (n = 8)		Curcumin (n = 6)	
	Pre	Post	Pre	Post
Systolic blood pressure, mmHg	110 ± 3	113 ± 3	112 ± 3	114 ± 2
Diastolic blood pressure, mmHg	60 ± 2	64 ± 3	64 ± 2	65 ± 2
Heart rate, beats/min	59 ± 3	61 ± 4	59 ± 2	58 ± 2
Brachial artery baseline diameter, mm	3.73 ± 0.15	3.69 ± 0.16	3.85 ± 0.18	3.85 ± 0.19
Brachial artery maximal diameter after cuff deflation, mm	3.96 ± 0.13	3.85 ± 0.14	3.98 ± 0.17	4.00 ± 0.17

Values are presented as mean ± SE.

**Figure 1.**
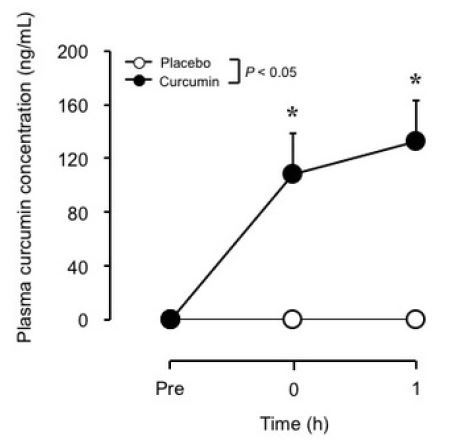
Changes (mean ± SE) in plasma curcumin concentration from baseline (Pre), immediately after exercise (0), and 1 h after exercise in the placebo and curcumin groups. *P < 0.05 vs. Pre.

No difference in plasma curcumin concentration at baseline was found between the curcumin and placebo groups, but a significant interaction effect was noted for the changes after exercise (Figure 1). Plasma curcumin concentration increased from 0.03 ± 0.02 ng/mL (baseline) to 108.6 ± 30.5 and 132.9 ± 29.9 ng/mL immediately and 1 h after exercise, respectively, in the curcumin group. However, in the placebo group, no significant changes in plasma curcumin concentration were observed from baseline (0.02 ± 0.02 ng/mL) at any time point after exercise (0.01 ± 0.01 and 0.02 ± 0.02 ng/mL immediately and 1 h after exercise, respectively).

**Figure 2.**
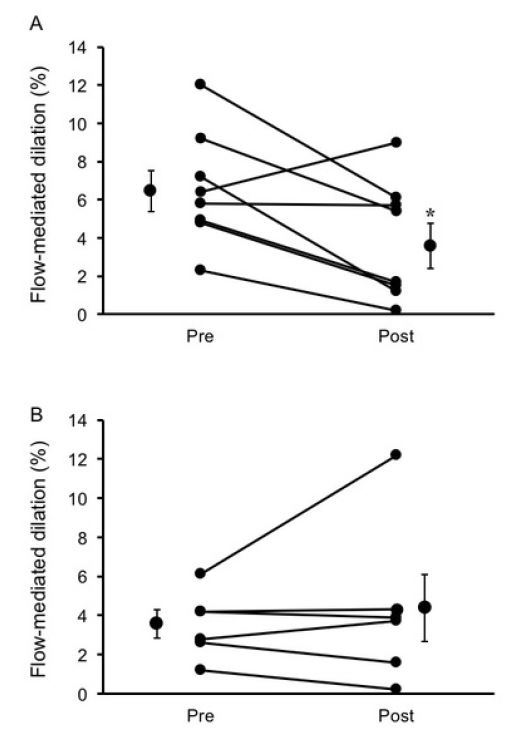
Individual subject percent flow-mediated dilation (FMD) responses at pre- and post-exercise time points in the placebo (A) and curcumin (B) groups. Mean pre- and post-FMD are expressed as mean ± SE in each group. *P < 0.05 vs. Pre.

**Figure 3.**
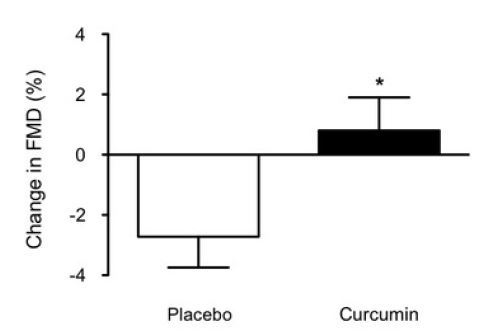
Changes in FMD responses after exercise in the placebo and curcumin groups. *P < 0.05 vs. placebo group.

A significant group × time interaction for FMD response was observed (*P* < 0.05). FMD significantly decreased in the placebo group (mean decrease, -2.7 ± 1.0%), whereas no change was observed in the curcumin group (0.8 ± 1.1%) (Figure 2). Furthermore, ANCOVA showed that the difference in FMD response between the groups was independent of baseline brachial artery diameter (*F* = 5.23, *P* < 0.05). The changes in FMD before and after eccentric exercise between the placebo and curcumin groups were significantly different (*P* < 0.05) (Figure 3).

## DISCUSSION

The major finding of the present study is that acute curcumin supplementation attenuated a decrease in endothelial function induced by eccentric exercise. We have already reported that acute eccentric exercise induced a decrease in endothelial function^9^. Therefore, we aimed to determine the acute vascular effect of curcumin ingestion on eccentric exercise-induced endothelial dysfunction. Our results suggest that acute curcumin supplementation with eccentric exercise would have a beneficial effect on exercise-induced endothelial dysfunction. 

In the placebo group, we noted a significant decrease in FMD after eccentric exercise (-2.7 ± 1.0%), an effect not observed at the same time point in the curcumin group. Our previous study and other studies have reported the beneficial effects of chronic curcumin ingestion on vascular endothelial function in healthy human subjects^14,15^. However, no information was available regarding its acute effect on eccentric exercise-induced endothelial dysfunction in humans. To the best of our knowledge, the present study is the first to provide evidence on the protective effect of acute curcumin supplementation against the decrease in endothelial function following eccentric exercise in healthy young men. 

FMD is a good indicator of vascular endothelial function, which reflects the bioactivity of endothelium-derived nitric oxide (NO). Several studies have reported that eccentric exercise is associated with elevated oxidative stress and inflammation^4,5,18^, which lead to decrease in endothelial function^6^. Superoxide anions (O2 ˙ ), one of the strong reactive oxygen species induced by eccentric exercise, scavenge endothelium-derived NO, as well as superoxide dismutase (SOD) in endothelial cells^19^. This considerably reduces the bioavailability of NO^20^, contributing to the decrease in endothelial function following eccentric exercise. 

A possible theory for the favorable effect of curcumin on eccentric exercise induced-endothelial dysfunction is that the antioxidant properties of curcumin could reduce oxidative stress in the vessel walls. Curcumin supplementation can attenuate exercise-induced oxidative stress by increasing blood antioxidant capacity^21^. Moreover, curcumin supplementation increased resting SOD activity^22^. Since O2 ˙ be normally dismutated by SOD in endothelial cells^19^, it leads to a considerable increase in the bioavailability of NO^20^. In addition to the antioxidative properties of curcumin, the attenuation of endothelial dysfunction following eccentric exercise may also be explained by the anti-inflammatory properties induced by curcumin supplementation. Several studies have reported that curcumin supplementation suppresses inflammation by downregulation of the expression of pro-inflammatory cytokines, such as TNF-α and IL-6^23^, which might contribute to its anti-inflammatory role in decreasing exercise-induced endothelial dysfunction. Taken together, these findings suggest that curcumin supplementation may attenuate the decrease in endothelial function by decreasing exercise induced-oxidative stress and inflammation. Further studies are needed to elucidate the mechanism underlying the effect of curcumin on eccentric exercise induced-endothelial dysfunction. 

In conclusion, acute curcumin supplementation significantly attenuated the decrease in endothelial function, as measured by FMD, following eccentric exercise in healthy young men. Our finding suggests that acute curcumin supplementation may have some beneficial effects on eccentric exercise-induced endothelial dysfunction.
